# Interaction between DNA and Drugs Having Protonable Basic Groups: Characterization through Affinity Constants, Drug Release Kinetics, and Conformational Changes

**DOI:** 10.3390/scipharm85010001

**Published:** 2017-01-04

**Authors:** Liliana P. Alarcón, Yolima Baena, Rubén H. Manzo

**Affiliations:** 1 Unidad de Investigación y Desarrollo en Tecnología Farmacéutica (UNITEFA), CONICET and Departamento de Farmacia, Facultad de Ciencias Químicas, Universidad Nacional de Córdoba, Córdoba X5000HUA, Argentina; lalarcon@fcq.unc.edu.ar; 2 Grupo de Investigación en Sistemas para Liberación Controlada de Moléculas Biológicamente Activas, Departamento de Farmacia, Facultad de Ciencias, Universidad Nacional de Colombia, Carrera 30 # 45-03, Bogotá D. C. 111311, Colombia; ybaenaa@unal.edu.co

**Keywords:** polyelectrolytes, DNA, drug interactions, complexation, physicochemical properties, circular dichroism, polymeric drug delivery systems

## Abstract

This paper reports the in vitro characterization of the interaction between the phosphate groups of DNA and the protonated species of drugs with basic groups through the determination of the affinity constants, the reversibility of the interaction, and the effect on the secondary structure of the macromolecule. Affinity constants of the counterionic condensation DNA–drug were in the order of 10^6^. The negative electrokinetic potential of DNA decreased with the increase of the proportion of loading drugs. The drugs were slowly released from the DNA–drug complexes and had release kinetics consistent with the high degree of counterionic condensation. The circular dichroism profile of DNA was not modified by complexation with atenolol, lidocaine, or timolol, but was significantly altered by the more lipophilic drugs benzydamine and propranolol, revealing modifications in the secondary structure of the DNA. The in vitro characterization of such interactions provides a physicochemical basis that would contribute to identify the effects of this kind of drugs in cellular cultures, as well as side effects observed under their clinical use. Moreover, this methodology could also be projected to the fields of intracellular DNA transfection and the use of DNA as a carrier of active drugs.

## 1. Introduction

The important biological relevance of the description of the structure and function of nucleic acids (NAs) (DNA and RNA), reported in the middle of the last century, led to a rapid expansion of studies concerning their physical, chemical, and biological properties [1]. Consequently, there is now vast literature providing a detailed description of many aspects of their molecular structures and related complex biological processes, which affect all living organisms [2].

With regard to the interaction of NAs with endogenous and exogenous organic molecules of molecular mass under 1000 g/mol [3,4]—a range in which a high proportion of active compounds currently used in therapy is located—many investigations have focused on the so-called intercalating molecules, given their importance in disrupting biological properties [5,6,7,8]. As a result, less attention has been paid to the study of the interactions of common drugs with NAs, despite the fact that during the administration of many dosage forms an appreciable concentration of the active compounds are in contact with living cells. 

An important proportion of water-soluble drugs contain basic groups that are protonated at physiological pH, and these drugs are able to interact as counterions of ionized phosphate groups of NAs. Related to this, there are reports dealing with the electrostatic interactions between NA phosphate groups and cationic lipids (forming so-called lipoplexes [9]) and also with molecules widely distributed in the body, such as spermine [10,11] and spermidine [12,13]. However, detailed information is not available on the many drugs that are able to ionically interact with NAs.

Phosphate groups of NAs (pKa regarded as 1.5 [1]) are ionized over a wide pH range, while hydroxyl groups present in guanidine and thiamine bases behave as weak acids (pKa 9.9 and 9.4 [1]). In addition, the moieties having protonable nitrogen atoms (present in adenine and cytosine) are also very weak basic centers (pKa 4.2 and 3.5 [1]). Therefore, phosphate groups are essentially ionized at pH values of physiological interest and confer the properties of anionic polyelectrolytes (PEs) to NAs with monomer units (nucleotides) of molecular mass ranging from 288.2 to 328.2 g/mol. In this regard, it is known that natural [14] and synthetic [15] acid PEs react with organic molecules having basic groups and generate a high proportion of ion pairs by counterionic condensation (Equation (1))

(1) $$\mathrm{RH}+D⇄R^{-}+DH^{+}⇄\left(R^{-}DH^{+}\right)$$

where RH, R^−^, *D*, *DH*^+^, and (R^−^*DH*^+^) represent an acid and an ionized group of the PE, the aqueous species of drugs, and an ion pair, respectively.

The aim of the present study was the characterization of the interaction of the phosphate groups of NAs with model drugs that have basic groups. Thus, this research was focused on obtaining information about the ability of NAs to form ion pairs R^−^*DH*^+^, drug effects on the structural properties of the macromolecule, and the reversibility of the interaction, together with the eventual contribution of other nonionic interactions. For this purpose, as the source of the NA we selected Parafarm^®^ DNA (Drogueria Saporiti S.A.C.I.F.I.A, Bs. As. Argentina) obtained from salmon sperm (DNA–Na) (widely used in cosmetic and nutritional research purposes [16,17,18,19,20]) and Sigma DNA (Sigma Chemical Co., St Louis, MO, USA) obtained from salmon sperm (denoted as DNA–Na^R^) as the reference. The set of drugs of recognized therapeutic utilities reported in Table 1 was selected to interact with DNA. 

The set contains highly lipophilic drugs such as propranolol (which exhibits high intestinal permeability and is essentially metabolized in liver [25]) and more hydrophilic drugs such as atenolol, whose permeability is lower and is mainly excreted by the kidneys [26].

## 2. Materials and Methods

### 2.1. Materials

Salmon sperm DNA–Na^R^ from Sigma Chemical Co. (St Louis, MO, USA; CAS No. 9007-49-2, salmon species *Oncerhynchus keta*, 41.2% G + C, highly polymerized [27]) and Salmon sperm DNA–Na from Parafarm^®^ (Drogueria Saporiti S.A.C.I.F.I.A, Bs.As. Argentina) were used. Hydrochlorides of atenolol, lidocaine, propranolol, and benzydamine and timolol maleate salts, all of pharmaceutical grade, were purchased from Parafarm. Sigma SYBR Green I (SIAL), agarose BioReagent for molecular biology, 1 Kb DNA Ladder, and PCR 100 bp Low Ladder were also used. A 10 mM solution of phosphate-buffered saline (pH 6.80; PBS) was prepared according to USP 34-NF 29 (2011) [28]. All other chemicals were of analytical grade, and Milli-Q water was utilized in the experiments. 

### 2.2. Analytical Determinations

Spectrophotometric determinations were obtained using the spectrophotometer Thermo-Electronic Corporation, Evolution 300 BB (Thermo-Electron Corporation, Rugby, England) with program PRO^TM^ Vision Software (Thermo-Electron Corporation), and the pH was determined at 25 °C using a pH meter Mettler Toledo Seven Multi, glass electrode Ag/AgCl DG 115-SC (Mettler-Toledo Sales Int. GmbH, Greifensee, Switzerland). 

The degree of neutralization of phosphate groups of DNA was obtained using differential scanning potentiometry (DSP), according to Luna et al. [29], while the proportion of Na^+^ in the samples was determined using the specific electrode perfectION^TM^ comb Na^+^ (Mettler-Toledo Sales Int. GmbH, Greifensee, Switzerlad). All titrations were carried out in duplicate.

Circular dichroism (CD) spectra of DNA and DNA–drug (DNA–D) complexes were obtained on a Jasco-810 spectropolarimeter (JASCO Corporation, Tokyo, Japan). The cell compartment was continuously purged with dry nitrogen, and data were recorded at a bandwidth of 2.0 nm, running speed 50 nm/min, response time 4 s, with measurements taken every 0.2 nm over 190–350 nm at 25 °C. A quartz cylindrical cell of 0.02 cm path length (Sigma Chemical Co) was used, which then was filled with 0.5 mL water and water–drug as blanks for correction of the DNA and DNA–D spectra, respectively. Each spectrum represented the average of three scans. The temperature cycle was produced using a circulating thermostated water bath (Haake^®^, Karlsrube, Germany), and Jasco CD spectropolarimeters recorded CD data as the ellipticity angle (Δε).

Electrokinetic potentials (ξ) were determined using a Delsa Nano C instrument (Beckman Coulter, Osaka, Japan) equipped with a 658 nm laser diode, a scattering angle set at 165°, temperature controller, and Delsa Nano 2.20 software (Beckman Coulter, Osaka, Japan). Measurements were taken in triplicate at 25 °C. Dispersions of DNA from 0.1% to 1.2% *w*/*v* alone and complexed with increasing proportions of drug were used.

The molecular mass of the DNA was determined by electrophoresis in a Mini-Cell GT Sub^®^ Cell (Bio-Rad Life Science, Beijing, China), as described previously by Sambrook et al. [30]. The samples were evaluated for duplicates in 0.75% agarose gel with SYBR Green (2 µL) in 1x TrisBorate-EDTA (TBE) buffer, and the electrophoresis was carried out at 100 V for 1.5 h. Finally, samples of DNA dissolved in loading buffer (12 µL) were seeded into the gel.

### 2.3. Preparation of DNA–Drug Complexes

The DNA–D*_x_* complexes were prepared by mixing appropriate volumes of aqueous solutions of the drug salts with dispersions of DNA–Na or DNA–Na^R^ previously hydrated for 24 h without agitation to obtain polymer concentrations of 0.60% or 0.12% *w*/*v*, respectively. The subscript “*x*” refers the molar proportion of drug with respect to the DNA phosphate groups, expressed as a percentage (i.e., *x* = 25%, 50%, 64%, and 85%). The dispersions were stored overnight at 4–8 °C before use.

### 2.4. Determination of Species Distribution and Affinity Constants (K)

The proportions of the *D*, *DH*^+^, and [$R^{-}DH^{+}$] species in the DNA–D*_x_* dispersions were determined by dialysis equilibrium using a tube of cellulose acetate membrane (12,000 Da; Sigma), according to a procedure described by Battistini et al. [31]. Exactly 10 mL of DNA–D*_x_* aqueous dispersions were put into the dialysis tube (donor compartment (*d*)), which was inserted in a stoppered flask (receptor compartment (*r*)) containing 100 mL (V*_d_*/V*_r_*: 1/10) or 400 mL (V*_d_*/V*_r_*: 1/40) of water for 24 h at 25 °C under magnetic stirring at 32 rpm. The pH of the *d* and *r* compartments was then recorded, and the drug concentration in the receptor ($\left[D\right]r=\left[DH^{+}\right]r+\left[D\right]r)$ was analyzed by absorbance spectroscopy at the maximum wavelength of each drug with reference to a standard curve prepared from drug solutions of known concentrations. The mass of drug in receptor compartment (D*r*) in mg was subtracted from the original mass (Dt) to obtain the mass of drug donor compartment (D*d*) (i.e., Dt − D*r* = D*d*) from which the dialysis ratio ([D]*r*/[D]*d*) was calculated. According to Equation (1), the species distribution in the D*d* can be calculated by the following equations:

(2) $$\frac{\left[D\right]r}{\left[D\right]d}=\frac{\left[DH^{+}\right]r+\left[D\right]r}{\left[D\right]d+\left[DH^{+}\right]d+\left[\left(R^{-}DH^{+}\right)\right]d}$$

(3) $$\mathrm{Ka}=\frac{\left[D\right]\left[H^{+}\right]}{\left[DH^{+}\right]}$$

The affinity constant for the counterionic condensation (Kcc) is given by [32]:

(4) $$\mathrm{Kcc}=\frac{\left[\left(R^{-}DH^{+}\right)\right]\left[H^{+}\right]}{\left[\mathrm{RH}\right]\mathrm{Ka}\left[DH^{+}\right]}$$

[RH] was calculated from Equation (5), in which [RH]_t_ represents the concentration of all phosphate groups of DNA.

[RH]t = [RH] + [(R^−^*DH^+^*)] + [R^−^] = [RH] + [(R^−^*DH^+^*)] + [*DH*^+^] (5)

Since the sum of the negative species $\left[R^{-}\right]$ plus $\left[{\mathrm{OH}}^{-}\right]$ and $\left[{\mathrm{Cl}}^{-}\right]$ is equal to the sum of the positive species $\left[DH^{+}\right]$ plus $\left[H^{+}\right]$ and $\left[{\mathrm{Na}}^{+}\right]$, and considering that $\left[{\mathrm{Cl}}^{-}\right]$ = $\left[{\mathrm{Na}}^{+}\right],\left[R^{-}\right]\gg \left[{\mathrm{OH}}^{-}\right]$ and $\left[DH^{+}\right]\gg \left[H^{+}\right]$, then $\left[R^{-}\right]$ approaches to $\left[DH^{+}\right].$

### 2.5. Drug Release from DNA–D_x_ Complexes

Release of drug from the DNA–D*_x_* complex dispersions was performed at 37.0 ± 0.1 °C in two-compartment Franz cells separated by a cellulose acetate membrane that had been previously hydrated in Milli-Q water for 30 min (D9527-100FT cellulose membrane dialysis tubing flat width 43 mm 12,000 Da; Sigma). In the donor compartment of each cell an accurately measured amount of the sample (1 mL) was introduced. The receptor compartment was filled with 16 mL of Milli-Q water, and 0.9 mL samples were taken at appropriate time intervals, with the volume removed replaced with fresh medium. The concentrations of drugs were determined by absorbance spectroscopy as previously described.

### 2.6. Reversibility of the Interaction 

The complexes of propranolol and benzydamine were subjected to exhaustive dialysis to recover uncomplexed DNA. To carry this out, samples of 10 mL of DNA–D_50_ were dialyzed following the methodology described above at a volume ratio of 1:40 (*d*/*r*) for 24 h, using 10 mM PBS (pH 6.8) as the receptor medium, which was replaced every two hours during the first eight hours. The donor dispersion, after being left overnight, was used for UV and CD determinations.

## 3. Results and Discussion

### 3.1. Preparation of Complexes of DNA with Model Drugs 

**Figure 1:**
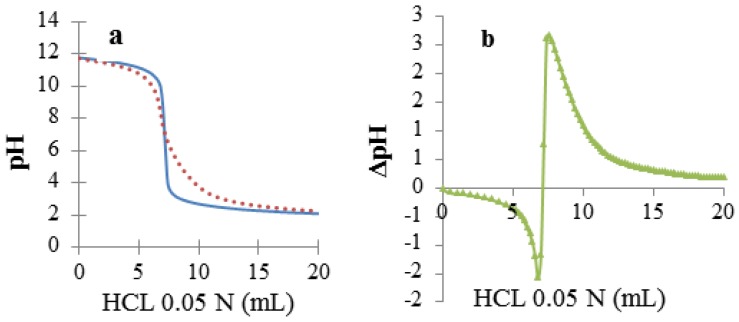
Differential scanning potentiometry (DSP) titration of DNA–Na^R^ (**a**) (—) NaOH vs HCl and (^....^) NaOH + DNA–Na^R^ vs HCl; and (**b**) ΔpH = (^....^) − (—) vs HCl.

**Figure 2:**
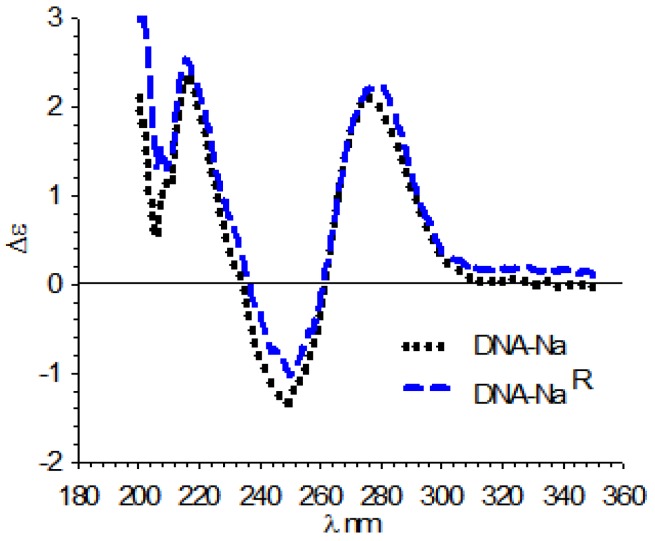
Circular dichroism (CD) spectra of DNA–Na^R^ and DNA–Na.

The aqueous dispersions of DNA–Na at 0.6% *w*/*v* and DNA–Na^R^ at 0.1% *w*/*v* used were first characterized with titration by DSP, which indicated that in both cases nearly all the phosphate groups were salified (Figure 1), and the potentiometric determination of Na^+^ revealed that DNA–Na contained 3.71 meq/g, and DNA–Na^R^ contained 3.51 meq/g of phosphate groups. Both samples exhibited a UV absorption maximum at 260 nm and absorbance ratios of A_260_/A_280_ near 1.8, indicating an acceptable degree of purity [33]. The CD of both samples showed a type-B secondary structure, which is the characteristic native structure of double-helix DNA [34,35,36] (Figure 2). On the other hand, gel electrophoresis indicated that DNA–Na^R^ exhibited a band between 2000 and 8000 base pairs, whereas that of DNA–Na corresponded to a size lower than 100 base pairs (Supplementary Materials).

The reaction of the aqueous dispersions of DNA–Na at 0.6% *w*/*v* with variable proportions of salts of the basic model drugs generated slightly viscous homogeneous dispersions. Similar results were also obtained with DNA–Na^R^ at 0.12% *w*/*v*. However, in this case, a lower concentration was used due to the viscosity produced by the high molecular mass (MM) of the macromolecule. Nevertheless, in both cases, on increasing the concentration of the complexes, the viscosity of the dispersions also increased, yielding translucent hydrogels that exhibited a good physical stability when stored for 20 days at 2–8 °C. Considering that the ionic equilibria are the main interactions involving phosphate groups of DNA and the protonated species *DH*^+^, Equation (1) can be replaced by:

(6) $$\mathrm{RNa}+DH^{+}A^{-}⇄R^{-}+DH^{+}{+A}^{-}⇄\left[R^{-}DH^{+}\right]{+\mathrm{Na}}^{+}{+A}^{-}$$

where R^−^ represents an ionized DNA phosphate and A^−^ represents the anion of the drug salts.

### 3.2. Species Distribution in the DNA–D Dispersions

The proportions of drug reversibly bonded to DNA in equilibrium with free species *D* and *DH*^+^ were determined through dialysis equilibrium using a semipermeable membrane that prevented DNA diffusion from the donor compartment. The time to reach the equilibrium (24 h at 25 °C) was determined by monitoring the evolution of the DNA–Pr_50_ system. Subsequently, a set of experiments was performed (reported in Table 2), in which the data obtained were processed according to Equation (6), as described above.

As shown in Table 2, a high fraction of drug that remained in the donor compartment was involved in ionic condensation with DNA. The model systems exhibited high DNA–D affinities, expressed as Kcc, which were of the order of 10^6^. The experiments at a volume ratio of 1/40 revealed that atenolol and propranolol exhibited similar Kcc values, which were higher than that of lidocaine, which had a lower pKa. This behavior was also observed previously with another acid PE [32].

During the dialysis, Na^+^ and Cl^−^, were able to freely diffuse to the receptor compartment to reach the equilibria in which they were involved. In this way, when the DNA–At_85_ system was subjected to dialysis with the addition of increasing proportions of NaCl, a concomitant lowering of both the remaining proportion of atenolol in the donor compartment and Kcc was observed. This behavior is evidence of competition between *DH*^+^ and Na^+^ to interact with the phosphate groups of DNA, as depicted by Equation (6). On the other hand, for the experiments at a volume ratio 1/10, the DNA–At*_x_* system, when loaded with higher proportions of atenolol, exhibited higher Kcc values than those loaded at lower atenolol proportions. Finally, Table 2 also shows that DNA–Na^R^ of high MM exhibited a similar behavior to that observed with DNA–Na.

### 3.3. Electrokinetic Potential (ζ)

The negative ζ of DNA–Na dispersions is highly concentration-dependent. For example, by increasing the concentration of DNA–Na from 0.6% to 1.2% *w*/*v*, the ζ values were lowered by 12 mV. The results reported in Table 3 were taken at the same concentrations employed in dialysis and kinetic determinations. Samples of DNA–Na at 0.6% and DNA–Na^R^ at 0.12% aqueous dispersions exhibited high negative ζ values of −28.04 and −35.86 mV, respectively, which were ascribed to the dissociation of the phosphate groups. Again, the higher concentrated dispersion exhibited the lower ζ.

As shown in Table 3, ζ decreased with an increase in the proportion of the loading drug, which was also observed with other acidic PEs [37] and is in line what the counterion condensation theory of PE that recognizes two extreme modes of counterion association, currently referred to as loose and covalent bonding [38,39,40].

### 3.4. Circular Dichroism (CD)

As mentioned above, it can be observed in Figure 2 that both samples of DNA–Na exhibited CD spectra characteristic of a double-helix secondary structure. The effect of temperature was shown by the reversible conversion towards the single secondary conformation. 

**Figure 3:**
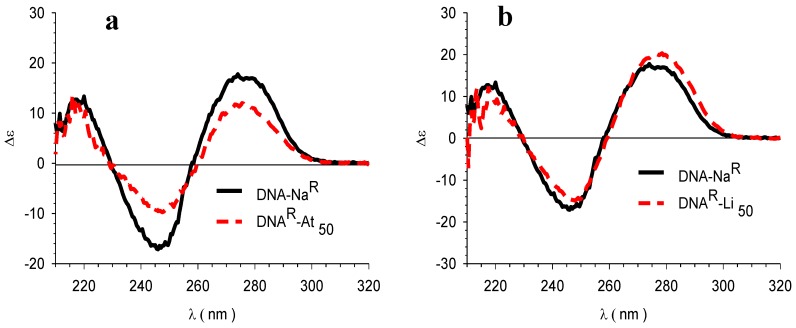

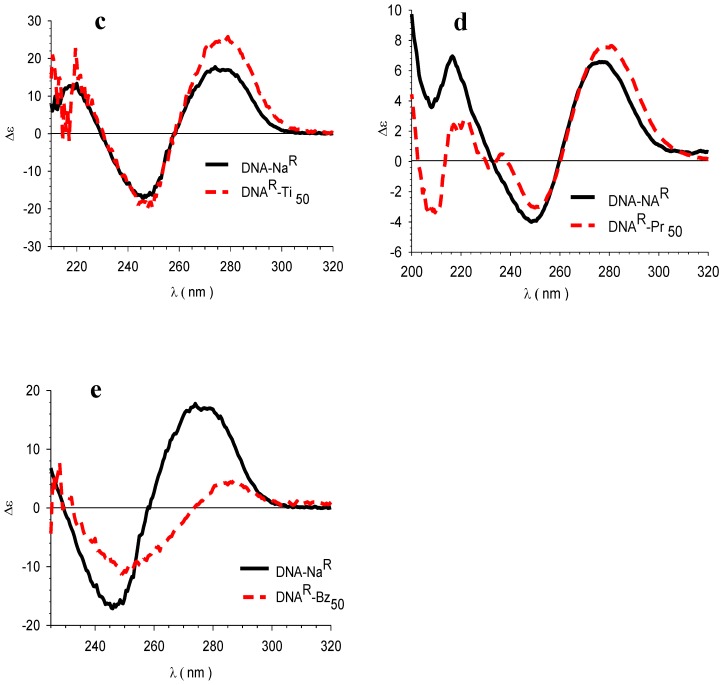
CD spectra of DNA–Na^R^ together with DNA^R^–D_50_ of the set of model drugs (**a**) atenolol (At), (**b**) lidocaine (Li), (**c**) timolol (Ti), (**d**) propranolol (Pr), and (**e**) benzydamine (Bz).

Complexes DNA–D_50_ with different models of drug exhibited differing CD profiles. In fact, those obtained with atenolol, lidocaine, and timolol (the drugs with the lowest lipophilicity) exhibited a positive Cotton effect at 280 nm, a negative one at 245 nm, and a crossing point at nearly the same wavelengths as native DNA, suggesting that they maintained the native double-stranded form of type-B secondary structure (Figure 3a–c). However, it was observed that the intensities of the signals in the DNA–At complexes decreased with greater atenolol loading (Supplementary Materials).

On the other hand, the profiles of the complexes with propranolol and benzydamine (the drugs of highest lipophilicity) suggested that the DNA did not maintain the type-B secondary structure, as shown in Figure 3d,e. In both cases, a significant reduction was observed in the negative signal with a redshift of the absorption maximum. In addition, the propranolol complex developed a greater intensity in the positive side, while that of benzydamine produced a decreased positive band that has previously been described as being a dehydrated form of the DNA [34,35,36,41].

**Figure 4:**
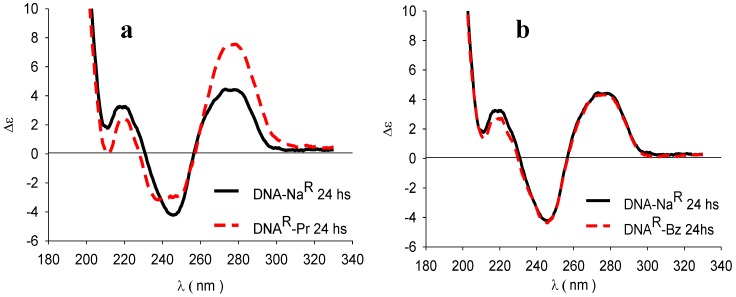
CD spectra of DNA–Na^R^ together with DNA^R^–D_50_ (drugs: (**a**) propranolol (Pr) and (**b**) benzydamine (Bz)) after exhaustive dialysis.

The dispersions of the propranolol and benzydamine complexes were subjected to an exhaustive dialysis in a buffer at pH 7.0. The recovered DNA of benzydamine dispersion exhibited the original native CD profile, but that of propranolol did not revert completely to the original profile (Figure 4a,b), with the propranolol recovered in the receptor media used, determined by UV spectrometry, only accounting for 88.2% of the original amount in the sample. This finding suggests a contribution of other nonionic interactions. Therefore, the changes observed in the secondary structure of DNA as a consequence of the interaction with the set of drugs appeared to be of a reversible nature in all cases except that of propranolol. 

### 3.5. Drug Release in Two-Compartment Diffusion Cells

**Figure 5:**
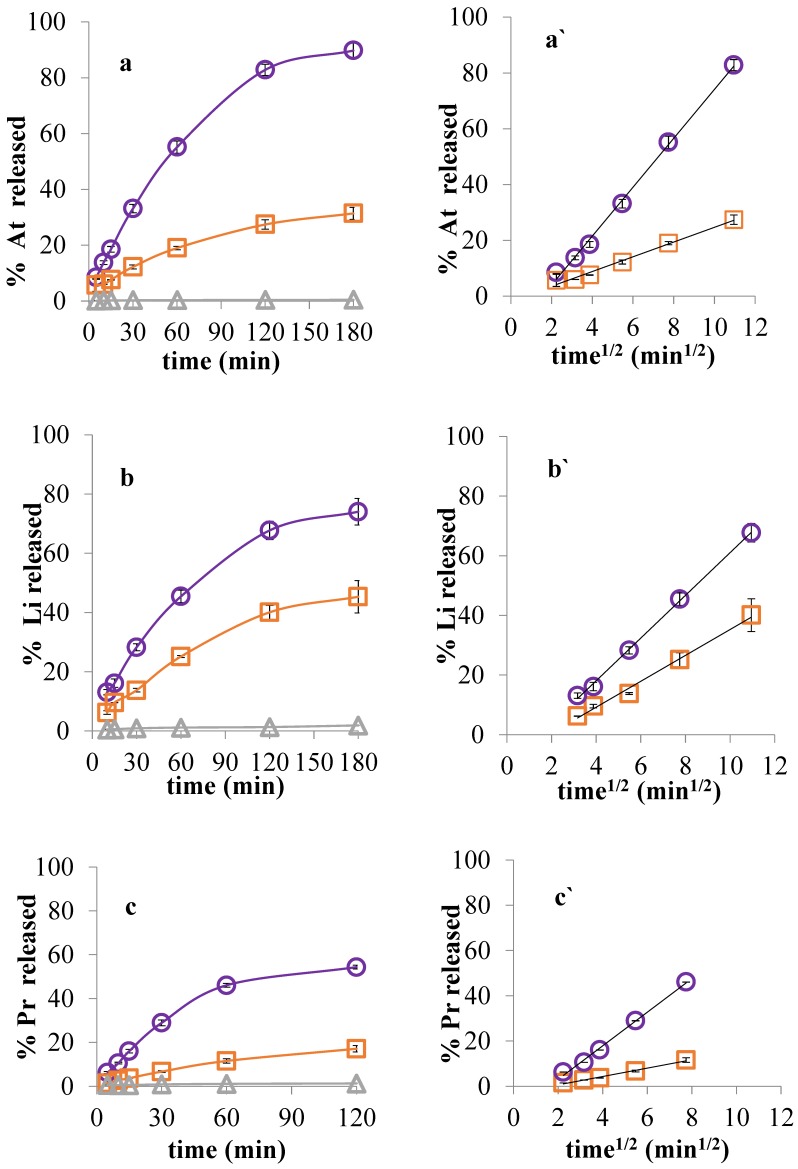
Cumulative drug release from both DNA–D_50_ (square) and free drug (circle) in Franz cells as a function of time (**a**–**c**) and time^1/2^ (**a’**–**c’**). DNA–NA (triangle) was included as a blank. Drugs: atenolol (At), lidocaine (Li), and propranolol (Pr).

The kinetics of drug release from dispersions of a set of complexes DNA–D*_x_* was investigated by placing them in the donor side of two-compartment cells bounded by a semipermeable membrane. The receptor compartment was filled with water, and the efficacy of the membrane to prevent the diffusion of the macromolecules was monitored by measuring the absorbance at 260 nm on samples of DNA–Na. Under these conditions, the reversible interaction DNA–D*_x_* generated a diffusion of the drug species towards the receptor compartment, with Figure 5a–c showing representative release profiles of three model drugs.

**Figure 6:**
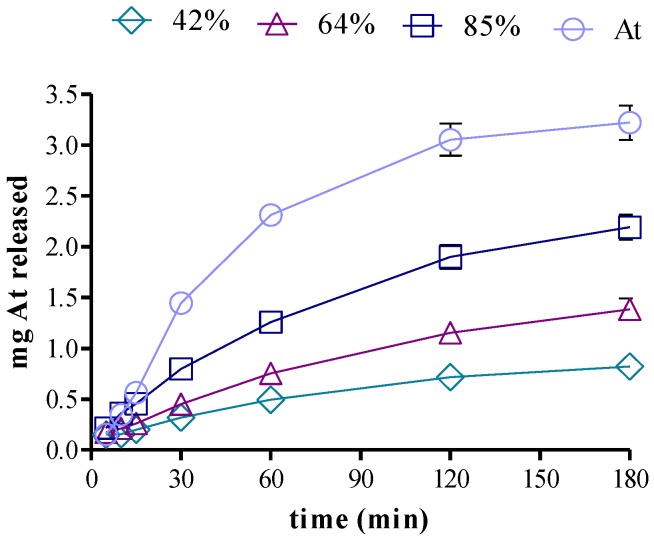
Cumulative atenolol (At) release from DNA–At*_x_* (*x*: 42% (rhombus), 64% (triangle), and 85% (square) and free At (circle) equivalent at 100%.

The rate of diffusion of the free species of drug was considerably slower than that of the reference drug placed in the donor compartment at an equivalent concentration, and this ability to modulate the release is consistent with the high degree of counterionic condensation previously observed. In fact, as depicted in Equation (6), both the uncharged *D* and the cationic *DH*^+^ together with A^−^ were able to freely diffuse and release, while (R^−^*DH*^+^) remained as a reversible drug reservoir. In line with these observations, the rates of drug release followed the classical Higuchi correlation with time^1/2^ [42], as can be seen in Figure 5a’–c’ and Table 4 that reports the regression parameters. It can be seen in the table that the slope of lidocaine release from the complex (4.33) is higher than those of atenolol (2.64) and propranolol (1.85) complexes, which is in accordance with the lower Kcc of lidocaine. This behavior is also evident from the comparison of the difference between the slopes of free and complex D (Δ slopes) reported in that table. The negative intercepts observed are consistent with the required time by the diffusion system to reach the kinetic quasi-equilibrium. Moreover, on keeping the concentration of DNA constant, while increasing the proportion of loading drug, a proportional increase in the drug release rate was observed, as shown in Figure 6. Accordingly, the systems behaved like carriers which slowly released drug, as observed with other similar PE–D systems [32,43].

The results presented in the preceding sections support the view that the main interaction between DNA and drugs with protonatable basic groups occurs through the DNA phosphate groups, thereby giving a high proportion of counterionic condensation, as depicted by Equation (6).

The high acidity of the phosphate groups resulted in the high affinity constants (Kcc) that were observed even in the presence of salts. In contrast, comparatively lower Kcc values were reported for PE with carboxylic pending groups of lower acidity [32]. The counterionic condensation (R^−^*DH*^+^) brought about a decrease in the electrokinetic potential, which did not compromise the physical stability of the dispersions.

In line with the reversibility of the DNA–D interaction, the drug release kinetics observed in the model systems assayed were consistent with a PE–D model previously developed [43,44], and no significant differences in the kinetic release behavior among the complexes assayed were observed.

Complexes of DNA with atenolol, lidocaine, and timolol did not exhibit modifications in the secondary structure of DNA. However, for the complexes with propranolol and benzydamine, the drugs of higher lipophilicity, a significant change in the conformation of DNA was observed. By performing exhaustive dialysis, the reversibility of DNA towards the original native conformation was observed in the case of benzydamine. However, for propranolol, no complete reversion to the original conformation was observed.

The drug interactions described here with DNA would also be expected to take place with other NAs, such as RNA. In addition, the in vitro behavior of NAs described above may occur in vivo during the pharmacokinetic sequence of events (Liberation, Absorption, Distribution, Metabolism, Excretion; LADME) originated by the administration of a dosage form containing a drug having basic groups. This is particularly so with drugs having high cellular membrane permeability such as propranolol, benzydamine, and lidocaine. Therefore, when these drugs penetrate and diffuse into living cells, they will be subjected to many interactions. Of these, the negative electrokinetic potential of NAs will attract the cationic species *DH*^+^, and their phosphate groups will become a target in which a strong electrostatic interaction takes place.

However, although it remains to be determined how such transient interactions would affect the functions of NA, the methodology described here to characterize in vitro DNA–D interactions through affinity constants and drug release rates together with their effects on the DNA conformation provides physicochemical basis to answer this question. Therefore, this methodology would contribute to identify effects of these kind of drugs in cell culture experiments to study cell proliferation, viability, and morphology as well as side effects observed under their clinical use. Besides, it could also be projected to the fields of intracellular DNA transfection [9] and the use of DNA as a carrier of active ingredients [45,46,47,48].

## Figures and Tables

**Table 1:** Physicochemical properties of the set model drugs.

Drug (D)	MM ^a^ (g/mol)	Type of Amine	pKa	Log PC o/w ^b^	Solubility (mg/mL)
Atenolol (At)	266.34	Secondary	9.54	0.22	13.3
Propranolol (Pr)	295.81	Secondary	9.53	3.48	7.09E^−2^
Timolol (Ti)	432.50	Secondary	9.53	2.12	2.74 ^c^
Lidocaine (Li)	234.34	Ternary	7.95	2.44	4.10 ^c^
Benzydamine (Bz)	345.90	Ternary	9.27	3.71 ^c^	5.11E^−2^

^a^ MM: Molecular mass of D; ^b^ Log PC o/w: partition coefficient logarithm in octanol/water. All data were taken from Avdeef A. Absorption and Drug Development (2003) [21], except those marked “^c^”, which came from PubChem [22,23,24].

**Table 2:** Species distribution at equilibrium and log Kcc obtained by dialysis of DNA–Dx complexes at 25 °C.

Ratio V*_d_*/V*_r_^a^*	Complex	D *^b^* Concentration (mg/mL)	D*_x_* (mol %)	pH Donor Equilibrium	Species distribution (%)			Log Kcc
					[R^−^*DH*^+^]	*DH^+^*	*D*	
1/40	DNA–At*_x_*	2.20	9.38	6.21	83.98	16.01	0.010	5.86
		3.33	18.34	6.31	83.28	16.70	0.010	5.79
		4.49	29.30	6.44	96.70	3.30	0.003	6.49
		6.60	28.99	6.19	91.49	8.51	0.004	6.30
	DNA–Pr*_x_*	1.25	11.25	5.66	76.06	23.94	0.004	6.06
		2.45	20.71	5.73	74.47	25.53	0.004	6.00
		3.72	24.51	5.72	69.78	30.21	0.005	5.92
		4.90	26.19	5.94	82.65	17.35	0.005	6.03
	DNA–Li*_x_*	2.24	9.42	5.91	85.01	14.75	0.230	4.51
		3.39	12.65	5.91	86.44	13.34	0.225	4.58
		4.48	20.59	5.81	84.94	14.87	0.191	4.66
	DNA–At_85_	4.48	28.79	6.44	96.70	3.30	0.003	6.49
	DNA–At_85_–NaCl_25_	4.48	22.67	6.18	91.19	8.81	0.004	6.26
	DNA–At_85_–NaCl_50_	4.48	14.08	6.29	89.98	10.01	0.006	6.04
	DNA–At_85_–NaCl_100_	4.48	13.32	5.99	49.35	50.63	0.018	5.37
1/10	DNA–At*_x_*	2.20	36.53	6.43	98.67	1.33	0.001	6.95
		3.33	59.44	6.41	99.27	0.73	0.001	7.42
		4.49	75.37	6.44	98.80	1.20	0.001	7.40
		5.52	92.46	7.00	97.22	2.77	0.009	6.98
	DNA^R^–At*_x_*	1.29	40.77	6.71	93.19	6.80	0.011	6.66
		1.57	41.95	6.63	86.60	13.39	0.018	6.43
	DNA^R^–Pr*_x_*	0.71	30.77	6.28	88.45	11.54	0.01	6.63
		1.45	43.60	6.25	63.24	36.74	0.02	6.09
		2.10	55.13	6.26	68.25	31.74	0.02	6.28
	DNA^R^–Li*_x_*	1.44	30.74	6.13	53.04	45.73	1.21	4.40
		2.13	50.93	6.07	70.29	29.03	0.69	4.93

*^a^* Volume ratio between donor and receptor compartments; *^b^* Concentration of drugs loaded in the complexes in the donor compartment before dialysis. The concentrations, in *w*/*v*, of DNA–Na and DNA–Na^R^ are 0.6% and 0.12%, respectively.

**Table 3:** Electrokinetic potential (ζ) (mV) of a set of DNA–D*_x_* complexes.

D Proportion (X mol %)	DNA–Na				DNA–Na^R^				
	At	Pr	Li	Me	At	Pr	Li	Ti	Bz
0	−28.04	−28.04	−28.04	−28.04	−35.86	−35.86	−35.86	−35.86	−35.86
25	−21.53	−21.24	−20.63	−18.96	-	-	-	-	-
45	−21.17	−19.62	−18.15	−18.50	-	-	-	-	-
65	−19.38	−19.16	−16.13	−14.53	−26.68	−34.91	−26.70	−28.06	−28.38
85	−16.59	−16.88	−15.60	−13.36	-	-	-	-	-

The concentrations, in *w*/*v*, of DNA–Na and DNA–Na^R^ are 0.6% and 0.12%, respectively.

**Table 4:** Slopes and y-intercepts from the Higuchi correlation of diffusion rates against time^1/2^ of D and DNA–D_50_, reported in Figure 5a’–c’.

System	Slope	Δ Slope	y-Intercept	*r*^2^
At	8.76	6.12	−13.56	1.000
DNA–At	2.64		−1.77	0.989
Li	7.15	2.82	−10.52	0.999
DNA–Li	4.33		−8.11	0.993
Pr	7.42	5.57	−11.80	0.996
DNA–Pr	1.85		−3.07	0.992

## Supplementary Materials

The following are available online at www.mdpi.com/2218-0532/85/1/1/s1. Figure S1: Agarose gel electrophoresis of DNA–Na was run on 1% agarose and visualized with SYBR Green I; Figure S2: Circular dichroism spectra of an aqueous dispersion of reference DNA–Na^R^ and DNA–At60–180 complex.
