# Supplementary material for: Interaction between DNA and Drugs Having Protonable Basic Groups: Characterization through Affinity Constants, Drug Release Kinetics, and Conformational Changes
Source: Sci Pharm. 2017 Jan 4;85(1):1. doi: 10.3390/scipharm85010001 (PMC5388140; doi:10.3390/scipharm85010001)
Supplement: Supplementary file 1 [file scipharm-85-00001-s001.pdf]

Supplementary Materials: Interaction between DNA and Drugs Having Protonable Basic Groups: Characterization through Affinity Constants, Drug Release Kinetics, and Conformational Changes

Liliana P. Alarcón, Yolima Baena and Rubén H. Manzo

a) b)

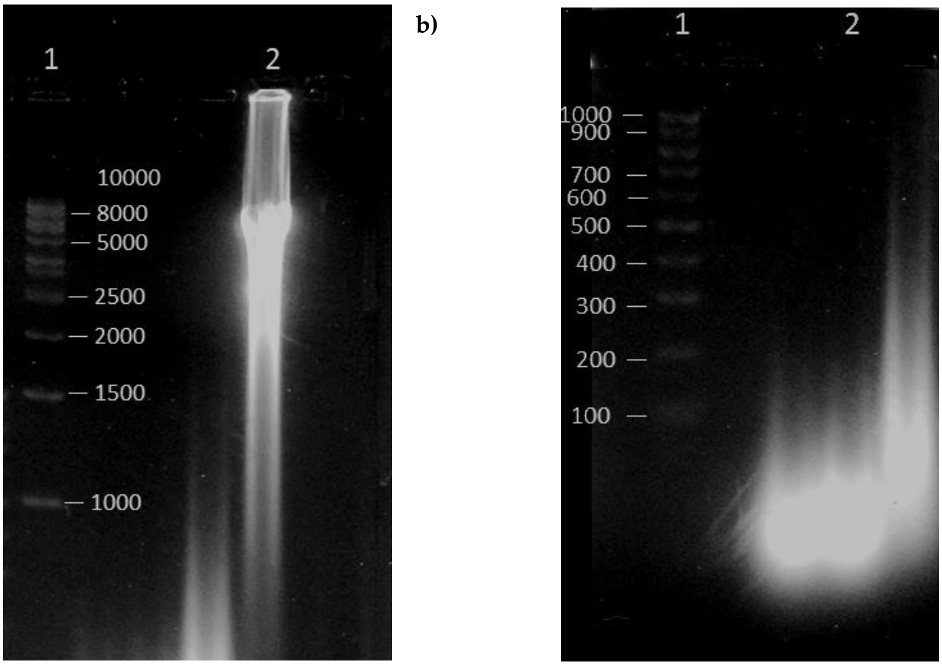

Figure S1. Agarose gel electrophoresis of DNA–Na was run on 1% agarose and visualized with SYBR Green I. a) lane 1 DNA size standard 1 Kb ladder; lane 2 DNA–Na^R. b) Lane 1 DNA size standard 100 bp low ladder; lane 2 DNA–Na.

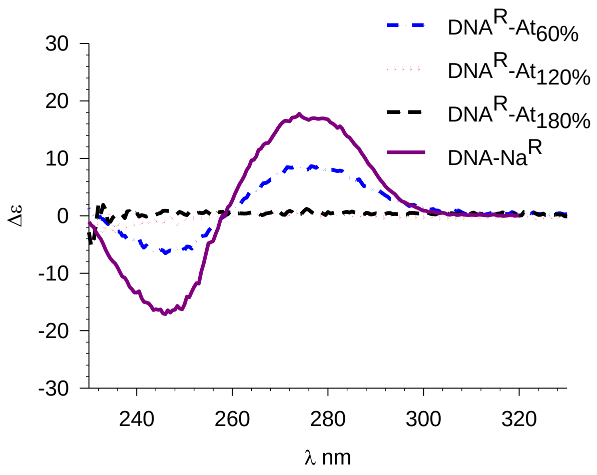

Figure S2. Circular dichroism spectra of an aqueous dispersion of reference DNA–Na^R and DNA–At$_{60–180}$ complex.
